# Supplementary material for: Telomere Function and the G-Quadruplex Formation are Regulated by hnRNP U
Source: Cells. 2019 Apr 28;8(5):390. doi: 10.3390/cells8050390 (PMC6562495; doi:10.3390/cells8050390)
Supplement: Supplementary file 1 [file cells-08-00390-s001.pdf]

Supplemental Figures

A

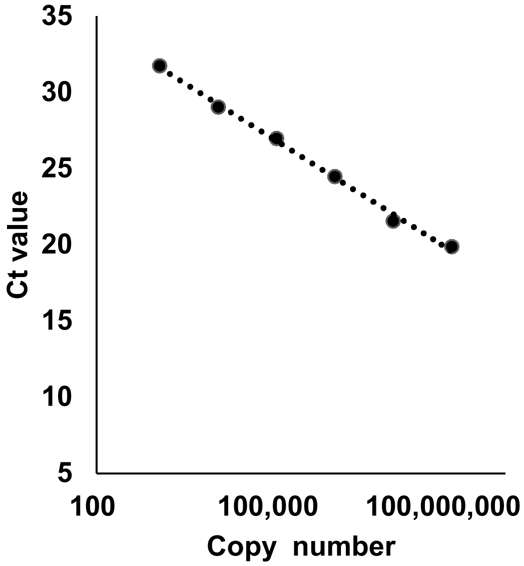

B

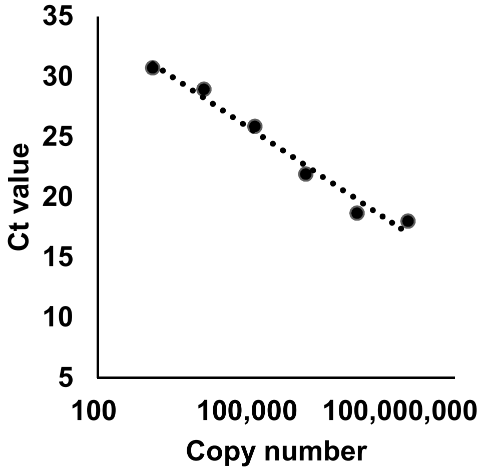

In order to generate a standard curve for qPCR, serial dilutions of oligonucleotide were made. The copy number of the oligonucleotide was calculated from (A) and (B) in Figures 4 and 5, respectively. X axes here expressed as logarithms.
